# Noninvasive Monitoring of Training Induced Muscle Adaptation with ^31^P-MRS: Fibre Type Shifts Correlate with Metabolic Changes

**DOI:** 10.1155/2013/417901

**Published:** 2013-07-15

**Authors:** Eike Hoff, Lars Brechtel, Patrick Strube, Paul Konstanczak, Gisela Stoltenburg-Didinger, Carsten Perka, Michael Putzier

**Affiliations:** ^1^Clinic for Orthopaedics, Center for Musculoskeletal Surgery and Julius Wolff Institute, Charité-Universitätsmedizin Berlin, 10117 Berlin, Germany; ^2^Berlin-Brandenburg Center for Regenerative Medicine, Charité-Universitätsmedizin Berlin, 13353 Berlin, Germany; ^3^Institute for Sports Medicine, Humboldt University of Berlin, 10115 Berlin, Germany; ^4^Institute for Cell and Neurobiology, Charité-Universitätsmedizin Berlin, 13353 Berlin, Germany

## Abstract

*Purpose*. To evaluate training induced metabolic changes noninvasively with ^31^P magnetic resonance spectroscopy (^31^P-MRS) for measuring muscle fibre type adaptation. *Methods*. Eleven volunteers underwent a 24-week training, consisting of speed-strength, endurance, and detraining (each 8 weeks). Prior to and following each training period, needle biopsies and ^31^P-MRS of the resting gastrocnemius muscle were performed. Fibre type distribution was analyzed histologically and tested for correlation with the ratios of high energy phosphates ([PCr]/[P_*i*_], [PCr]/[**β**ATP] and [PCr + P_*i*_]/[**β**ATP]). The correlation between the changes of the ^31^P-MRS parameters during training and the resulting changes in fibre composition were also analysed. *Results*. We observed an increased type-II-fibre proportion after speed-strength and detraining. After endurance training the percentage of fast-twitch fibres was reduced. The progression of the [PCr]/[P_*i*_]-ratio was similar to that of the fast-twitch fibres during the training. We found a correlation between the type-II-fibre proportion and [PCr]/[P_*i*_] (*r* = 0.70, *P* < 0.01) or [PCr]/[**β**ATP] (*r* = 0.69, *P* < 0.01); the correlations between its changes (delta) and the fibre-shift were significant as well (delta[PCr]/[P_*i*_] *r* = 0.66, delta[PCr]/[**β**ATP] *r* = 0.55, *P* < 0.01). *Conclusion*. Shifts in fibre type composition and high energy phosphate metabolite content covary in human gastrocnemius muscle. Therefore ^31^P-MRS might be a feasible method for noninvasive monitoring of exercise-induced fibre type transformation.

## 1. Introduction 

In 1873, Ranvier described a classification of human skeletal muscle fibres, classing them as red, slow-twitch or white, fast-twitch. In the slow-twitch fibres, commonly classified as type-I, metabolism is predominantly oxidative whereas, in the fast-twitch type-II-fibres, major production of energy is of glycolytic character. Knowledge of fibre type distribution and adaptation are still important in sports medicine, rehabilitation, and clinical environment [1–6]. Fibre type composition and its adaptation to specific training seem to be a crucial factor to predict and monitor athletic performance [7–9]. To date, the gold standard for measuring fibre type composition and transformation in human skeletal muscle is the histological evaluation of needle biopsy tissue samples. This technique is associated with several drawbacks. The small tissue sample may not be representative for the whole muscle and is perhaps just a single snapshot of the resting muscle [10]. Due to the technical challenges and its invasiveness, biopsies are usually not performed repeatedly in longitudinal studies or under dynamic conditions.

As proven in animal experiments, ^31^P magnetic resonance spectroscopy (^31^P-MRS) enables a noninvasive and repeated measurement of the concentrations of high-energy phosphates, which have been shown to be different in slow- and fast-twitch single fibres or in muscles which are dominant for one fibre type [11–14]. ^31^P-MRS studies in humans demonstrated that athletes with obviously different fibre type composition (e.g., sprinters and long distance runners) feature different ^31^P resonance spectra of the resting leg muscles [15–19]. So far, publications correlating biopsy results with the ^31^P-MRS measurements in resting human muscles are heterogeneous [17, 20, 21]. Vandenborne et al. [17] and Takahashi et al. [21] have shown that ^31^P-MRS might be a feasible method to evaluate fibre type composition in the vastus lateralis, gastrocnemius, and soleus muscles. Both found a correlation between the type-II-fibre proportion and different ratios of the high energy phosphates whereas Kuno et al. [20] observed no correlation at all. This inconsistency is likely owed to the differences in the methods used [22]. Another reason could be that both the fibre type distribution as well as the phosphorous metabolite content are heterogeneously distributed within the human muscle and show a large degree of interindividual variance [23, 24]. Therefore it might prove more precise to determine the intraindividual changes of these metabolites in correlation to specific muscle adaptation. To our knowledge, there are no published data, reporting on the longitudinal investigation of training induced fibre type adaptations measured by ^31^P-MRS.

The purpose of our study was to identify ^31^P-MRS parameters for practical application which reasonably predict a predominance of a fast or slow fibre type composition in the medial human gastrocnemius muscle by modifying the ^31^P-MRS experimental setup using a conventional 1.5 Tesla tomograph. Additionally, we analyzed the feasibility of monitoring fibre type adaptation to regimes of speed-strength, endurance, and detraining employing a correlation analysis between ^31^P-MRS and biopsy.

## 2. Material and Methods

### 2.1. Subjects

Eleven male healthy volunteers (sports students) were enrolled in the present study. Six of the subjects were endurance trained (road cycling, long distance running) and three were speed-strength trained athletes (sprint, volleyball); the other three participants had no history of regular specific training. Anthropometric as well as endurance and strength related characteristics are shown in Table 1. Muscle biopsies were taken and ^31^P magnetic resonance spectra were acquired before (*t*_0_) and after each of three cycles of training regime (*t*_*S*_, *t*_*E*_, *t*_*D*_), which had been intended to induce muscle fibre type transformation. The subjects were on a normal diet and were not permitted strenuous activities during the weeks leading up to the experiments. All subjects were informed about the purpose of the study and their written consents were obtained. The local ethics committee approved the research protocol, which is demonstrated in Table 2.

### 2.2. Training Interventions

Regimes of speed, strength, endurance, and detraining were carried out over a period of 24 weeks (Table 2). Subjects refrained from any additional lower body resistance or endurance training throughout the study. After the baseline measurements, training began with speed-strength regimes. During an eight-week-period the subjects underwent a training protocol of seven ballistic countermovement jump and apparatus exercises specific for the calf muscles with three sessions per week, each separated by at least 24 hours of recovery. Training volume and intensity started with submaximal loads and were increased progressively up to 100%, with no more than one repetition. The endurance training consisted of an eight-week running program with three sessions in the first three weeks and the remaining four sessions in the following five weeks. Training duration and intensity were incrementally adapted to the individual anaerobic threshold (IAT) (between 70 and 100%) as monitored by heart rate. During the eight weeks detraining period subjects were requested to avoid particular sports training. All workloads above daily routine had to be documented. The training protocols described above were designed to invoke specific adaptations of muscle structure [2, 25–28] especially fibre type shifts with increasing type-II-percentage after speed-strength and detraining or type-I-proportion after endurance training.

### 2.3. ^31^P Magnetic Resonance Spectroscopy Measurements

All measurements were performed using a Siemens Magnetom Vision (Siemens, Erlangen, Germany) whole body scanner with a superconducting magnet at 1.5 T, with a corresponding resonance frequency of 63.9 MHz for ^1^H and 25.9 MHz for ^31^P, respectively. The spectra were collected using a 7 cm diameter surface coil, placed directly above the muscle belly. This small coil allows the almost exclusive acquisition of signals of the medial part of the gastrocnemius muscle after optimizing the signal depth of indentation by adjusting the pulse parameters using a phantom according to Takahashi et al. [21]. The phantom consisted of a two-layer agarose gel—of similar consistency as human musculature—with a 0.1 molar phosphate buffer of two different pH: pH 5.5 in the superficial 1 cm layer and pH 6.4 in the deep 4 cm layer. We varied the pulse amplitude while keeping all other pulse parameters (e.g., pulse duration) constant to minimize the signal from the superficial layer. For the repeated measurements, relative positions of the coil to bony landmarks of the lower leg were noted, to regulate the measurement conditions. Magnetic field homogeneity was optimized by maximizing the proton signal of intramuscular water. Water half linewidths were lower than 10 Hz. Acquisition parameters were 1024 data points, 1000 Hz sweep width, a transmitter voltage of 25 V, and an amplification of the receiver of 105 dB. Phosphorous spectra resulted from the sum of 128 free induction decays (FIDs) using a pulse-acquire sequence with a rectangular pulse of 500 *μ*s duration and no localization method. Total measurement time was 6 : 24 min with a pulse repetition time of 3 s. The number of acquisitions and the repetition time were determined from pilot testings as compromise between measurement time and saturation effects [29, 30]. The employed repetition time of 3 s is shorter than T1 of the observed metabolites at 1.5 T, especially for PCr (T1 for 1.5 to 3 T = 5.0 to 6.6 s, as reviewed by Meyerspeer et al. [31]). Comparing relaxed (TR = 8 s) with partially relaxed spectra (TR = 1 – 7 s) we observed a saturation effect of 2–12.8% when employing a repetition time of three seconds. These results were used for T1 correction of our spectra. In our opinion, this is acceptable since the aim of our study was the implementation of a robust and applicable method in a clinical setting or in sports medicine for examining patients and probands at rest and during dynamic load in the tomograph.

### 2.4. MRS Data Analysis

The acquired data vector was expanded to 2048 data points by zerofilling. Prior to FFT a Lorentzian line broadening with 2 Hz was applied. The absorption spectra were obtained using zero and first order phase correction. Before quantification the baseline was corrected with a polynominal fit routine. Individual peak areas were determined using an in-house developed software. These areas correspond to the concentration of phosphomonoester (PME), inorganic phosphate (P_*i*_), phosphodiester (PDE), phosphocreatine (PCr), and ATP. For all metabolites instead of PCr a Lorentzian line shape was assumed. For the PCr-peak we used a combination of Lorentzian and Gaussian line shape. The doublets *α* and *γ*ATP were fitted as sum of two, and the triplet *β*ATP as a sum of three separately fitted peaks. With the iterative fit algorithm the difference of the area under the baseline corrected spectrum and the fitted curve was minimized. A representative spectrum and the corresponding fit are shown in Figure 2. Ratios of [PCr]/[P_*i*_], [PCr]/[*β*ATP], and [PCr + P_*i*_]/[*β*ATP] were subsequently calculated. Differences (delta) of these ratios between the measurement points were calculated as well; an increase over time was indicated by a positive value and a decrease over time by a negative value.

To assess spectral quality chemical shift, half width and signal-to-noise ratios, and standard deviation of theses parameters were calculated for both the PCr- and P_*i*_-resonance. The half width was defined as the full-width at half maximum of PCr, respectively, P_*i*_. SNR was defined as the ratio between the PCr- and P_*i*_-peak amplitude and the peak-to-peak spectral noise, in the range of +10 to +20 ppm.

### 2.5. Muscle Biopsy

**Figure 1:**
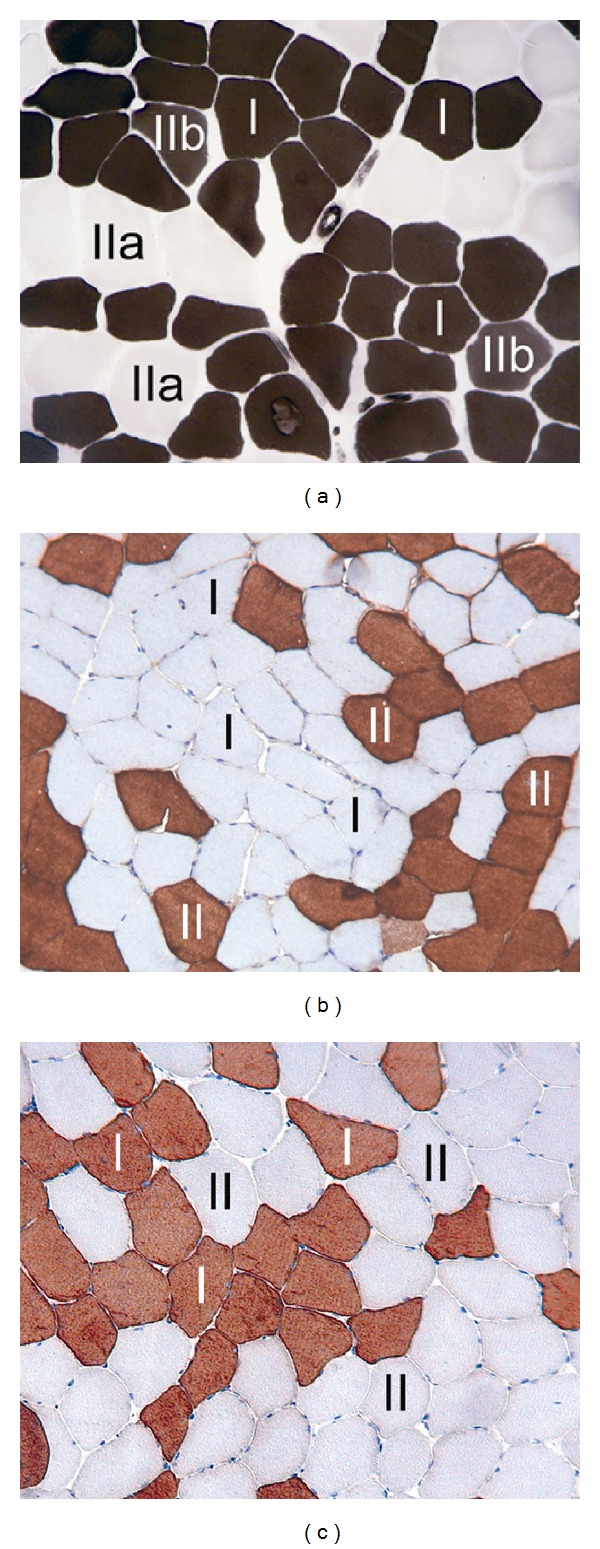
Biopsy samples of the gastrocnemius muscle showing examples for fibre type classification utilizing different staining methods—(a) ATPase enzymohistochemistry preincubation at pH 4.6 (classified into slow-twitch type I and fast-twitch type IIa/IIb), (b) MHC_fast_ immunohistochemistry, (c) MHC_slow_ immunohistochemistry (classified into slow-twitch type I and fast-twitch type II with MHC staining).

**Figure 2:**
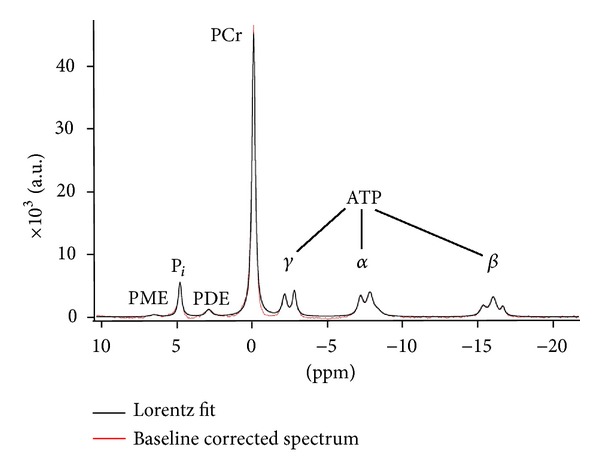
Representative ^31^P-MRS spectrum of the human calf musculature (red) and Lorentz fit (black). All spectra were acquired on a 1.5 T whole-body scanner with a 7 cm diameter surface coil. A rectangular pulse-acquire sequence was used (pulse duration = 500 *μ*s; TR = 3 s; 128 acquisitions). PME: phosphomonoester, P_*i*_: anorganic phosphate, PDE: phosphodiester, PCr: phosphocreatine, ATP: adenosine triphosphate, a.u.: arbitrary units.

Tissue samples were obtained from the medial part of the right gastrocnemius muscle using the percutaneous needle biopsy technique (biopsy apparatus: Bard, Covington, GA, USA; diameter: 2.5 mm; penetration depth: 22 mm). One or two days after each MRS measurement a biopsy was obtained, so that a total of four biopsies were taken from each subject in the present study. Tissue samples were mounted and frozen rapidly in isopentane precooled with liquid nitrogen. For further histological and histochemical *in vitro* experiments we used established protocols for transverse sectioning and staining (ATPase, MHC_fast_, MHC_slow_) as described by Bancroft and Gamble [32]. Fibre types were classified into type I and type II (all staining methods), as well as the subtypes IIa and IIb (ATPase reaction only) (Figure 1). The average fibre diameter was determined for a minimum of 200 individual fibres per section. Muscle fibre composition was calculated as a percentage value (%), and as relative cross-sectional area for each fibre type (% area). Mean values from two independent observers were calculated and statistically analyzed. Differences (delta) in percentage value and relative cross-sectional area between the measurement points were calculated as well; an increase over time was indicated by a positive value and a decrease over time by a negative value.

### 2.6. Statistical Analysis

A nonparametric one-way-ANOVA for repeated measures (Friedman test) followed by Dunns *post hoc* tests was used to examine the impact of training on the parameters of muscle fibre type adaptation and on the relative content of high-energy phosphates. To determine training specific effects a Wilcoxon test was performed for these parameters to compare the single measurement points: *t*_0_ versus *t*_*S*_, *t*_*S*_ versus *t*_*E*_, and *t*_*E*_ versus *t*_*D*_. A parametric bivariate analysis (Pearson's *r*) was performed to test for correlation between muscle fibre type and the relative contents of the high-energy phosphates ([PCr]/[P_*i*_], [PCr]/[*β*ATP], [PCr + P_*i*_]/[*β*ATP]) and the changes (delta) of these parameters resulting from the training. Testing for normality of distribution (Kolmogorov-Smirnov-Test) had been carried out before. A nonparametric bivariate analysis (Spearman's *ρ*) was performed to test for the correlation between the changes of these parameters between the single measurement points, owing to the small sample size. Interobserver variability was examined employing *κ*-statistics. Statistical analyses were performed using the SPSS 17.0 statistics software (SPSS Inc., Chicago, IL, USA) and GraphPad Prism 5 (GraphPad Software Inc., San Diego, CA, USA).

## 3. Results

At *t*_0_^31^P-MRS spectra and muscle biopsies could be obtained from all volunteers and analyzed. At *t*_*S*_ two biopsy samples could not be used for analysis because of tissue damage due to thawing. At *t*_*E*_ we lost two volunteers who withdrew their consent for further needle biopsies. All volunteers documented a strict execution of the training protocol as demanded. Overall 38 of 44 possible pairs of ^31^P-MRS spectra and muscle biopsies (86.4%) and 26 of 33 possible pairs for the calculation of changes between the measurement points (78.8%) were available for evaluation.

**Figure 3:**
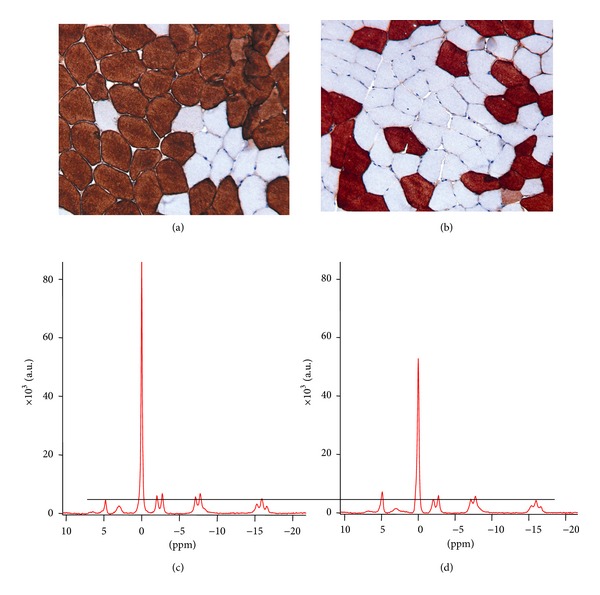
Representative cross section of a biopsy sample (MHC_fast_ immunohistochemistry) (a) and the corresponding ^31^P-MRS spectrum (c) of a subject predominant for type-II-fibres and the biopsy sample (b) and spectrum (d) of a subject predominant for type-I-fibres. The spectrum of the subject predominant for the fast-twitch fibres (c) shows a higher concentration of PCr and a lower concentration of P_*i*_ compared to the spectrum of the subject predominant for slow-twitch fibres (d) assuming a constant ATP concentration of 8.2 mM [11, 22, 33, 34] as indicated by the black horizontal line. (Both datasets are scaled the same), a.u.: arbitrary units.

Figure 3 shows ^31^P-MRS spectra obtained from 2 volunteers and their corresponding cross section of the tissue biopsy. The figure demonstrates the predominance of type-II-fibres in one volunteer and type-I-fibres in the other. At all measurement time points we found a wide range of the fibre type proportion (14.7 to 78.2% type-II-fibres) and the spectroscopic parameters (Table 3). The results of the histological methods (ATPase and MHC staining) correlated positively (*r* = 0.98; *P* < 0.01). The interobserver *κ* was 0.97.

**Figure 4:**
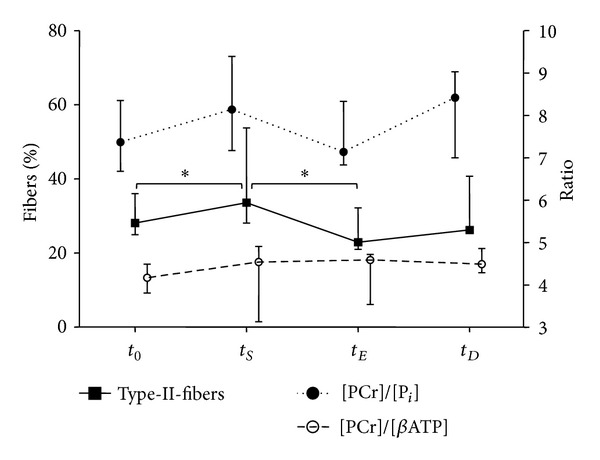
Changes in the distribution of type-II-fibres, [PCr]/[P_*i*_] and [PCr]/[*β*ATP] during intervention. Training induced changes of type-II-fibres were similar in pattern to those of [PCr]/[P_*i*_] but different to those of [PCr]/[*β*ATP]. Parameters are shown as median values; whiskers indicate interquartile range (IQR); Asterisk (∗) indicates *P* < 0.05. Friedman test was not significant for the 3 parameters during intervention; Wilcoxon test resulted in a significant increase in type-II-fibres between *t*_0_ and *t*_*S*_, and in a significant decrease between *t*_*S*_ and *t*_*E*_ (*P* < 0.05). *t*_0_: pretraining, *t*_*S*_: after speed-strength training, *t*_*E*_: after endurance training, *t*_*D*_: after detraining.

**Figure 5:**
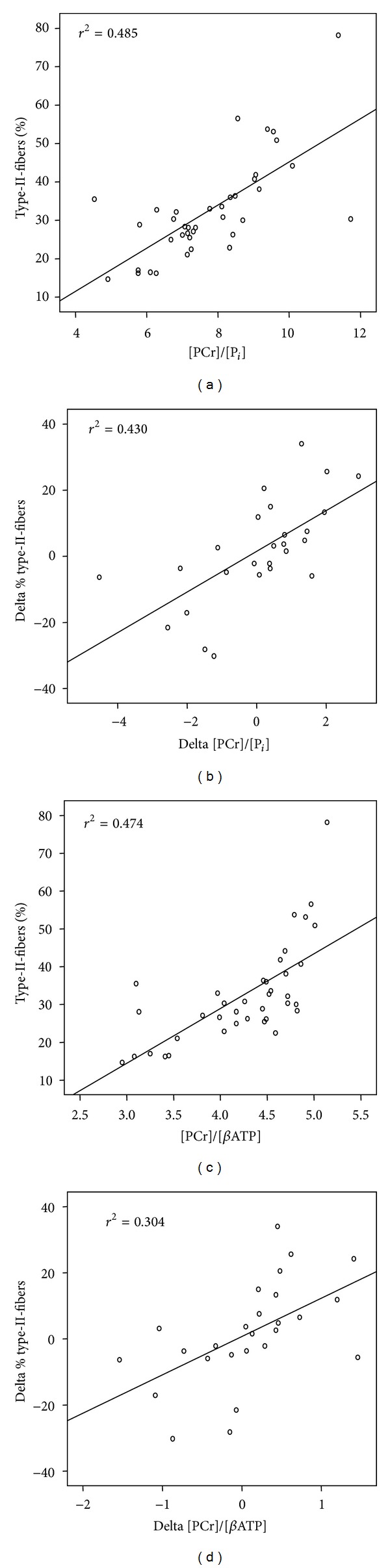
Scatter plots for percentage (%) type-II-fibres and [PCr]/[P_*i*_] (a), or [PCr]/[*β*ATP], respectively, (c), as well as differences in percentage (delta %) type-II-fibres between the measurement points and differences in [PCr]/[P_*i*_] (delta[PCr]/P_*i*_]) (b), or [PCr]/[*β*ATP] (delta[PCr]/[*β*ATP]), respectively, (d). 38 of 44 (86.4%) evaluable pairs of ^31^P-MRS and biopsy data were available for the calculation of (a) and (c), 26 of 33 (78.8%) for (b) and (d) throughout the 4 measurement points. The relationship between the parameters and the strength of correlation is indicated by the line and by the correlation coefficient (*r*²).

Type-II-fibres increased in proportion (%) and proportional area (% area) after speed-strength and detraining and decreased after endurance training (one-way-ANOVA statistically not significant; Wilcoxon test for *t*_0_ versus *t*_*S*_ and *t*_*S*_ versus *t*_*E*_ *P* < 0.05). The training induced changes of type-II-fibres were similar in pattern to those of [PCr]/[P_*i*_] (increase after speed-strength and detraining; decrease after endurance training) but different to those of the [PC]/[*β*ATP]-quotient (as demonstrated in Figure 4 and Table 3). A positive correlation was found between the proportion and the percentage area of type-II-fibres and the [PCr]/[P_*i*_]- (%: *r* = 0.70, *P* < 0.01; % area: *r* = 0.66, *P* < 0.01) and [PCr]/[*β*ATP]-ratios (%: *r* = 0.69, *P* < 0.01; % area: *r* = 0.62, *P* < 0.05). No correlation was observed between fibre type proportion and [PCr + P_*i*_]/[*β*ATP]-ratio. We observed a positive correlation of the changes in proportion and percentage area of type-II-fibres during the training to the changes in [PCr]/[P_*i*_]-(%: *r* = 0.66, *P* < 0.01; % area: *r* = 0.54, *P* < 0.05) and [PCr]/[*β*ATP]-ratios (%: *r* = 0.55, *P* < 0.01; % area: *r* = 0.52, *P* < 0.05). These correlations were based on all 26 pairs of evaluable changes (delta) in ^31^P-MRS and biopsy independent from the training stimulus (Figure 5). Focussing on the different training interventions, the correlation analysis revealed a positive correlation only after the endurance training period (delta *t*_*S*_ to *t*_*E*_) between fibre type adaptation and the [PCr]/[P_*i*_]-ratio (%: *ρ* = 0.79, *P* < 0.05; % area: *ρ* = 0.78, *P* < 0.05).

The results of the parameters indicating the spectral quality are shown in Table 4. The variance was largest for the signal-to-noise ratio, but minimal values were greater than ten for the P_*i*_-resonance.

## 4. Discussion 

The purpose of the present study was to evaluate ^31^P-MRS as a noninvasive method for the monitoring of fibre type adaptation in human muscle *in vivo*. Additionally, we tried to identify ^31^P-MRS parameters which might reasonably predict a predominance of a fast or slow fibre type composition in a clinical setup. With our methodical approach we found that [PCr]/[P_*i*_]- and [PCr]/[*β*ATP]-ratio are closely related to the type-II-fibre proportion. To our knowledge we are the first to show ^31^P-MRS to be a feasible method for determining and monitoring training-induced muscle fibre adaptations noninvasively.

The findings of the present study regarding exercise-induced fibre type composition and fibre shifts closely match those previously published [2, 5, 26–28, 35]. We found a slightly lower proportion of type-II-fibres than described for the gastrocnemius muscle in other studies [17, 36]. The high proportion of endurance-trained subjects in our study group could be an explanation for this. We observed large interindividual differences in fibre type adaptation after the same training stimulus, which could be attributed to the heterogeneity of the participants' basal fibre type compositions and the differences in individual adaptability of the skeletal muscles [5].

The positive correlation between type-II-fibre proportion and ratios of [PCr]/[P_*i*_] and [PCr]/[*β*ATP] is consistent with that in several studies involving athletes with obviously different fibre type composition. For resistance trained subjects with high percentage of type-II-fibres such as sprinters [15, 37] or downhill skiers [18], elevated ratios of [PCr]/[P_*i*_] and [PCr]/[*β*ATP] have been reported. Conversely, endurance trained subjects which are predominant for type-I-fibres such as long-distance runners [15, 19, 37] or cross-country skiers [18] exhibited reduced values of these parameters. Furthermore, in several animal experiments a correlation between biopsy and ^31^P-MRS was observed [13, 38]. Human studies showed inhomogeneous results [17, 20, 21]. Takahashi et al. [21] described a good correlation of the type-II-fibre proportion to the [PCr]/[*β*ATP]-, but not to the [PCr]/[P_*i*_]-ratio for the vastus medialis muscle, whereas Vandenborne et al. [17] found opposing results for the lateral gastrocnemius and soleus muscle. Kuno et al. [20] reported no correlation at all for the vastus medialis muscle. These inconsistent results possibly relate to differences in the methods employed [22]. Takahashi et al. [21] used an unlocalized acquisition technique with a phantom-optimized small surface coil, similar to the one employed in our study. However, the data were the sum of only 32 FID, a fourth of the acquisitions we used. As a result from this, spectral resolution was inferior to that of the present study. The peaks of PME, P_*i*_, PDE, PCR, and *γ*ATP conflate which precludes the calculation of the single metabolites and therefore the accuracy of the [PCr]/[P_*i*_]-ratio. This has less effect on the [PCr]/[*β*ATP]-ratio. The localized technique used by Vandenborne et al. [17] leads to a considerable variability in spectral quality, especially in terms of the signal-to-noise ratio. Therefore, the values for metabolites with small concentrations, for example, [*β*ATP] and [P_*i*_] were affected markedly, resulting in increased [PCr]/[P_*i*_]- and [PCr]/[*β*ATP]-ratios. Additionally, this study group reported of a change of the ^31^P measurement parameters throughout their study. However, they found a moderate correlation between the type-II-fibre proportion and the [PCr]/[P_*i*_]-ratio. Due to the high relative standard errors reported by Vandenborne et al. [17] and studies using a similar technique [39], the correlation found between fibre type composition and [PCr]/[*β*ATP]-ratio was not statistically significant. The variability owing to the different techniques was equal to the difference in PCr-concentration between type-I- and type-II-fibres, with constant *β*ATP-concentration in the different fibre types in human muscle [11, 22, 33, 34]. Kuno et al. [20] used a large surface coil combined with a nonlocalized acquisition technique. It is therefore likely that signals from neighbouring muscles with different fibre type compositions were collected. Clearly, this has a big influence on the ^31^P-MRS data, contributing to the nonsignificance of their tested correlation. In recent studies with improved techniques especially for localized acquisition of ^31^P spectra the above-mentioned problems in signal-to-noise-ratio, temporal, spectral, and spatial resolution, described in older studies comparing ^31^P-MRS with biopsy, were solved [22, 40, 41]. Therefore, localized spectra might be even more precise than our results because contaminating signals from other muscles can be eliminated [41]. However, these works were conducted utilizing a higher field strength which is currently not available as standard for measurements in a whole-body scanner in daily use, which was the focus of our study. Although we found good correlations between the biopsy and ^31^P-MRS data, the results should be interpreted carefully, especially with respect to possible practical applications. Due to methodical limitations (e.g., short TR and the usage of relative concentrations as described in greater detail in the methods section) the calculated ratios of [PCr]/[P_*i*_]- and [PCr]/[*β*ATP] are not quantitative but rather of qualitative nature. Furthermore, the absolute values we found cannot easily be transferred to other MRS scanners. Additionally, the heterogeneity of the phosphorous metabolite content within human skeletal muscle at rest and during exercise is known from other studies using chemical shift imaging [23, 24]. Therefore we believe that a precise prediction of fibre type composition based only on the relative concentrations of unlocalized ^31^P spectra is not adequate. But the method seems to be feasible to predict a predominantly slow or fast fibre composition.

Additionally, we observed that training induced fibre type transformation correlates with a shift in the basal content of phosphocreatine and inorganic phosphate in human skeletal muscle when assuming a constant concentration of ATP at rest of 8.2 mM as previously described [11, 22, 33, 34]. We did not quantify the absolute concentrations of the metabolites from the biopsies or with ^31^P-MRS [11, 34]. Therefore we can only speculate about the changes of (absolute) concentration of the single metabolites. The higher correlation of fibre type adaptation to changes in [PCr]/[P_*i*_]-compared to that of [PCr]/[*β*ATP]-ratio implicates that the change in P_*i*_-concentration is larger than the change in [PCr] in the context of fibre type adaptation. These findings are supported by data published on the crucial influence of P_*i*_-concentration on force generated in the skeletal muscle. While a reduction in the basal P_*i*_-concentration is associated with an increased maximum isometric force [42], high basal P_*i*_-levels in slow muscles may increase the muscle's ability to maintain activation at the expense of peak-force [13]. In mammalians pure slow-twitch muscles contain only half of the [PCr] but a 3- to 8-fold concentration of inorganic phosphate [13]. In humans these differences are much smaller. PCr-concentration differs by 15–20% between type-I- and -II-fibres, [P_*i*_] by 100–300% [11]. Compared to these results from animal trials, this is a possible explanation for the weaker correlation found in our present study. Furthermore, it explains our finding that ^31^P-MRS in humans is not sensitive enough to detect small differences in muscle fibre transformations. The observed correlations were significant for the whole study period and for *t*_*E*_ ([PCr]/[P_*i*_]) when looking only at a single training period, despite the interindividual variability. Directly after the speed-strength training with an increasing type-II-fibre proportion, endurance training induced the exact opposite stimulus on fibre type transformation and metabolic adaptations. Noninvasive monitoring of fibre type adaptation with ^31^P-MRS in humans is possible only with an adequate transformation in combination with a high quality and low variability of the spectroscopic data. Otherwise the detectable differences *in vivo* would be smaller than the measurement error of the technique itself. A combination with dynamic measurements to calculate the skeletal muscle oxidative capacity with ^31^P-MRS might be helpful to further differentiate but is very time dependant and requires an MR-compatible ergometer, which is not commonly available for clinical use [22, 43].

^31^P-MRS of the resting muscles seems to be a useful method, particularly for longitudinal observation in sports medicine. Furthermore it might be reasonable as an additional tool in the diagnosis and monitoring of (neuro) muscular and metabolic diseases. The method's potential in talent screening could not be estimated because our results should not be uncritically transferred to children. The monitoring of fibre type adaptations in highly trained subjects with low transformation rates on the basis of changes of the basal high-energy phosphate concentrations does not seem to be possible. Large adaptations, for example, in untrained persons, after trauma or long rest periods during recreation, may be monitored accurately and noninvasively with ^31^P-MRS. Therefore, this method seems to be very useful for evaluating different training protocols in healthy subjects and patients as well as during patient rehabilitation.

Admittedly, our study is not without limitations. For example, only the medial gastrocnemius muscle was investigated. Whether the findings can be translated to other muscles has to be proven in future studies. On the other hand, tissue biopsy may be obtained from almost every muscle [36], ^31^P-MRS' quality strongly depends on muscle volume and localization. The technique described in the present study delivers good and stable results for a superficial leg muscle of large volume. Therefore, we believe our method to be feasible for the examination of comparable extremity muscles, prerequisite further validation. Additionally, it might be possible to adopt our technique for muscles of smaller volume or muscles in a deeper layer employing localized acquisition of ^31^P spectra. Furthermore we did not quantify the absolute concentrations of the metabolites. This would potentially allow inferring more on the biophysical effects which directly or indirectly cause the changes in observed metabolite quantities. These techniques had not been standardized for measurements of subjects in a whole-body scanner at the time we initiated our study but have been demonstrated to be feasible at rest and even during exercise in more recently published studies [34, 40, 41]. Beside this, the results of the present study must be interpreted carefully since the parameters which are based on peak values calculated from NMR resonance spectra are also partially influenced by factors other than metabolite concentration (e.g., relaxation effects). Furthermore, it seems with our chosen sweep width and digital block size that even with a zero-filled dataset the resolution may be at risk of undersampling, especially for the narrowest (and largest) peak, the phosphocreatine. We addressed this potential error using a combination of Lorentzian and Gaussian line shape for this peak. Nevertheless, because of the quality and constance of the spectra observed we conclude the influence of the last-mentioned factors within the present study to be quite negligible. Due to hardware component specific influences and factors such as differences in measurement parameters or different analytical software, the comparability with other studies is somewhat limited for ^31^P-MRS. Finally, an alternative noninvasive method, the tensiomyography based solely on biomechanical parameters, has been described, which allows the determination of the myosin heavy chain I proportion in superficial human skeletal muscle [44]. The muscle contraction is not exclusively dependent on the myosin heavy chain but also the myosin light chain distribution and particularly the overall enzyme activity [6]. Therefore we suppose that ^31^P-MRS is favourably to reflect these biochemical aspects especially the changes during an adaptation process [5, 6].

## 5. Conclusion

In conclusion, we have shown that ^31^P-MRS is a feasible noninvasive technique for determining training-induced adaptations in the human calf muscle. Therefore, it is a powerful tool for a longitudinal investigation in clinics, rehabilitation, and sports medicine. Further investigation employing the now available enhanced acquisition techniques will possibly substantiate its value for the described potential applications.

## Figures and Tables

**Table 1:** Patient anthropometric endurance and strength related characteristics.

	MV ± SD	Min	Max
Age (years)	27.9 ± 2.1	25.2	30.8
Body height (cm)	181.4 ± 6.5	173.0	194.0
Body weight (kg)	80.9 ± 8.7	64.3	92.1
BMI	24.5 ± 1.7	20.9	27.2
Rel. VO_2peak _ (mL/kg BW)	51.0 ± 5.4	45.5	62.4
IAT (% *v*_max⁡_)	76.1 ± 4.7	71.4	85.6
*F*_max⁡_ plantar flexion (N)	270.4 ± 22.8	229.2	287.5
*F*_iso_ 500°/s (N)	56.2 ± 7.7	50.6	72.1

MV: mean value. SD: standard deviation. MIN: minimum. MAX: maximum. BMI: body mass index. rel. VO_2peak_: maximal O_2_ uptake relative to body weight (BW). IAT: individual anaerobic threshold. *F*_max⁡_: maximal isometric power during plantar flexion. *F*_iso_: maximal isokinetic power during plantar flexion at 500°/s.

**Table 2:** Study design.

Time	Research/training
*t*_0_	Spiroergometry/speed-strength tests ^31^P-MRS (7 days after sports tests) Biopsy (within 2 days after ^31^P-MRS)

Week 1–8	Speed-strength training

*t*_*S*_-week 9	^ 31^P-MRS (5 days after last training) Biopsy (within 2 days after ^31^P-MRS)

Week 10–17	Endurance training

*t*_*E*_-week 18	^ 31^P-MRS (5 days after last training) Biopsy (within 2 days after ^31^P-MRS)

Week 19–26	De-training

*t*_*D*_	^ 31^P-MRS Biopsy (within 2 days after ^31^P-MRS)

**Table 3:** Relative contents of the high energy phosphates and the fibre type distribution for the 4 measurement points. ^31^P-MRS measurements and biopsies were performed initially (*t*_0_) and after a period of speed-strength (*t*_*S*_), endurance (*t*_*E*_), and de-training (*t*_*D*_). Fibre types were classified into slow-twitch more oxidative type I and fast-twitch more glycolytic type II with subtypes IIa and IIb (ATPase reaction).

	*t*_0_			*t*_*S*_			*t*_*E*_			*t*_*D*_		
	Median ± SE	Min	Max	Median ± SE	Min	Max	Median ± SE	Min	Max	Median ± SE	Min	Max
MRS												
[PCr]/[P_*i*_]	7.37 ± 0.51	4.91	9.16	8.14 ± 0.50	5.76	9.56	7.14 ± 0.49	6.10	10.09	8.42 ± 0.64	6.27	11.38
[PCr]/[*β*ATP]	4.17 ± 0.21	2.95	4.70	4.54 ± 0.31	3.08	4.97	4.59 ± 0.22	3.45	4.82	4.49 ± 0.21	3.41	5.14
[PCr + P_*i*_]/[*β*ATP]	4.80 ± 0.14	4.26	5.35	5.01 ± 0.23	3.61	5.52	5.29 ± 0.23	3.82	5.55	5.11 ± 0.25	3.94	5.73

Biopsy												
Type I (%)	71.9 ± 6.26	33.0	85.3	66.4 ± 5.89	43.5	83.8	77.1 ± 3.48	55.8	83.5	73.8 ± 7.75	21.8	83.8
Type II (%)	28.1 ± 2.98	14.7	38.1	33.6 ± 5.89	16.2	56.5	22.9 ± 3.48	16.5	44.2	26.2 ± 7.75	16.2	78.2
Type IIa (%)	23.6 ± 3.30	12.4	37.4	32.0 ± 4.83	15.7	48.4	22.1 ± 2.62	11.9	34.0	23.5 ± 6.54	15.7	65.5
Type IIb (%)	2.0 ± 0.65	0.0	5.1	5.3 ± 1.90	0.5	11.7	5.2 ± 1.26	0.5	10.2	1.9 ± 1.72	0.5	12.7

SE: standard error. MIN: minimum. MAX: maximum. *t*_0_: preinterventional. *t*_*S*_: after speed-strength training; *t*_*E*_: after endurance training. *t*_*D*_: after de-training.

**Table 4:** Chemical shift (CS), half width (HW), and signal-to-noise ratio (SNR) for the PCr- and P_*i*_-resonance.

	CS	±SD	HW	±SD	SNR	±SD	Min
P_*i*_	4.92	0.06	8.36	1.18	16.9	6.24	11.1
PCr	0*	—	6.37	0.74	148.4	47.49	86.0

*The chemical shift of PCr was set to zero.

SD: standard deviation; MIN: minimum.
